# Selective CB_2_ Agonist *JWH-133* Suppresses Viability and Migration-Related Responses in Prostate Cancer Cells

**DOI:** 10.3390/ph19071114

**Published:** 2026-07-19

**Authors:** Seda Sabah Özcan, Rehime Yapar, İsmail Değerli, Levent Elmas, Mehmet Korkmaz, Murat Çakır

**Affiliations:** 1 Department of Medical Biology, Faculty of Medicine, Manisa Celal Bayar University, Manisa 4500, Türkiye; seda.ozcan@cbu.edu.tr (S.S.Ö.);; 2 Department of Medical Biology, Faculty of Medicine, İzmir Bakırçay University, Izmir 35660, Türkiye; 3 Department of Physiology, Faculty of Medicine, Yozgat Bozok University, Yozgat 66200, Türkiye

**Keywords:** cannabinoid receptor type 2, *JWH-133*, prostate cancer, cell viability, migration, UN SDG 3: Good Health and Well

## Abstract

**Background/Objectives**: Cannabinoid receptor type 2 (CB_2_) agonists have attracted attention because of their potential effects on tumor-related cellular processes in different cancer models. In the present study, we investigated the effects of the selective CB_2_ agonist *JWH-133* on prostate cancer cell lines (LNCaP, DU-145, and PC3). **Methods**: Cell viability was evaluated using the MTT assay, while colony-forming capacity and migration-related responses were assessed by colony formation and wound-healing assays, respectively. In addition, the expression levels of selected cell-cycle- and apoptosis-related genes were analyzed by quantitative real-time PCR. **Results**: *JWH-133* reduced cell viability in a time- and concentration-dependent manner, although the magnitude of this effect differed among prostate cancer cell lines. The compound also reduced colony formation in PC3 and LNCaP cells and decreased wound closure in PC3 cells under the experimental conditions used. Furthermore, *JWH-133* altered the expression of several cell-cycle- and apoptosis-related genes in DU-145 and PC3 cells. **Conclusions**: Overall, these findings suggest that *JWH-133* modulates multiple cellular responses in prostate cancer cells in a cell-line-dependent manner. However, additional mechanistic studies are required to clarify the molecular pathways underlying these effects.

## 1. Introduction

Prostate cancer is the second most common malignancy among men worldwide, accounting for an estimated 1,466,680 new cases and 396,792 deaths in 2022 [1]. The incidence of prostate cancer varies considerably across geographic regions and population groups, displaying a strong correlation with advancing age. While only 1 in 350 men under the age of 50 are diagnosed with prostate cancer, this rate escalates to 1 in 52 among men aged 50 to 59, with the prevalence reaching approximately 60% in individuals aged 65 and older [2]. Current therapeutic options encompass prostatectomy, cryotherapy, radiation therapy, hormonal therapy, chemotherapy, and immunotherapy. Despite the clinical success of these protocols, the emergence of resistant phenotypes severely compromises disease prognosis, leaving limited therapeutic avenues for castration-resistant prostate cancer cells [3]. Furthermore, the adverse side effects associated with conventional therapies present significant challenges in patient management [4,5]. Consequently, the development of novel and improved therapeutic strategies remains of paramount importance.

The endocannabinoid system consists of cannabinoid receptors, cannabinoid ligands, and enzymes that synthesize and degrade cannabinoid ligands. Cannabinoid receptors (CB), which are G-protein-coupled receptors (GPCRs), have two distinct types: the cannabinoid type 1 receptor (CB_1_) and the cannabinoid type 2 receptor (CB_2_). CB receptors regulate various physiological states and cellular functions, including cell proliferation, migration, and survival [6,7]. The CB_1_ receptor, which is associated with the psychoactive effects of cannabinoids, is highly expressed in the brain. CB_2_, which is more widely distributed in peripheral tissues, is not associated with cannabinoid psychoactivity; therefore, it may be more advantageous to utilize the therapeutic effects of cannabinoids that act as selective CB_2_ agonists [8]. Cannabinoids have been shown to modulate several cellular processes pivotal to tumor progression, including proliferation, migration, survival, angiogenesis, and immune regulation, highlighting their relevance in oncology research [9]. Notably, CB_2_ expression levels are significantly elevated in human prostate cancer tissues compared to normal prostate epithelial cells [10]. Previous reports indicate that the downregulation or pharmacological inhibition of CB_2_ enhances prostate cancer cell viability, whereas CB_2_ activation exerts robust antiproliferative effects [11]. While many cannabinoid receptor ligands exhibit little or, at best, moderate selectivity for both receptors (CB_1_ and CB_2_), *JWH-133* exhibits 200-fold selectivity for the CB_2_ receptor [12].

Despite accumulating evidence suggesting that cannabinoid receptor signaling influences prostate cancer progression, the precise effects of selective CB_2_ agonists across diverse prostate cancer cell phenotypes remain incompletely understood.

However, prior studies have demonstrated the antiproliferative and antimigratory effects of cannabinoid receptor activation in various cancer models [12,13,14]; the responses of androgen-dependent and androgen-independent prostate cancer lines to selective CB_2_ activation may vary owing to their distinct molecular profiles and biological behaviors. To address this gap, the present study investigated the effects of the selective CB_2_ agonist *JWH-133* on cell viability, colony formation, migratory responses, and the expression of key cell-cycle- and apoptosis-related genes in LNCaP, DU145, and PC3 prostate cancer cell lines, representing different molecular and biological characteristics of prostate cancer. While LNCaP cells are androgen-sensitive and express functional androgen receptors (AR), DU145 and PC3 cells are androgen-independent, and PC3 cells further display a highly metastatic phenotype together with PTEN deficiency.

## 2. Results

### 2.1. Cytotoxic Activity/MTT Assay

**Figure 1:**
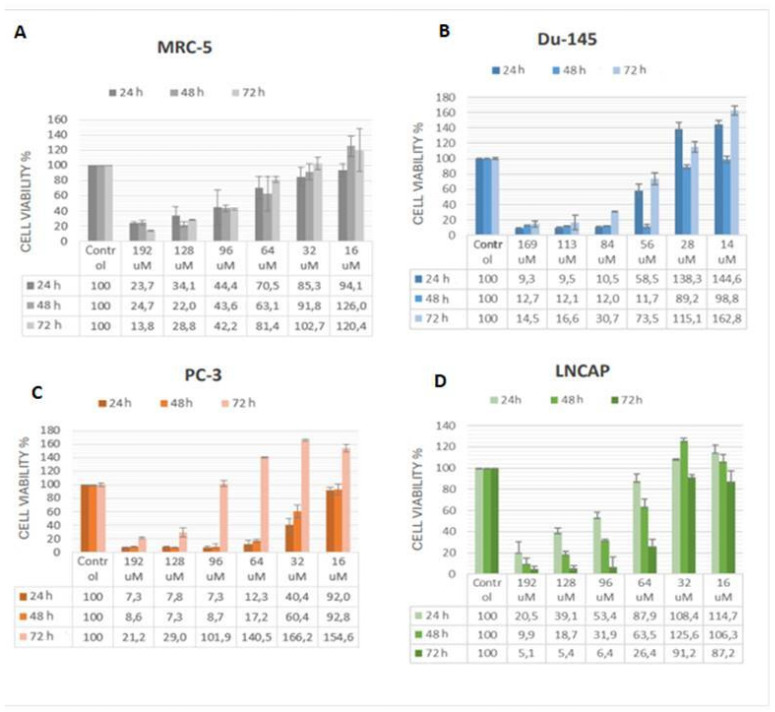
**Effect of *JWH-133* on the viability of MRC-5 (control) (A), Du-145 (B), PC-3 (C), and LNCaP (D) cells.** The cells were treated with *JWH-133* at different concentrations and time intervals, and their proliferation was assessed by the MTT assay. Data shows the average results of three independent experiments. IC_50_, the half-maximal inhibitory concentration.

The effects of *JWH-133* on cell viability were evaluated using the MTT assay in MRC-5, DU-145, PC3, and LNCaP cells following 24, 48, and 72 h of treatment. *JWH-133* reduced cell viability in a time- and concentration-dependent manner in prostate cancer cell lines. Among the tested cells, PC3 cells exhibited the highest sensitivity to *JWH-133* treatment, with an IC_50_ value of 24.87 μM at 24 h. In contrast, IC_50_ values for DU-145 and LNCaP cells were determined as 36.62 μM and 66.68 μM at 48 h, respectively (Figure 1). In the present study, MRC-5 cells were used as a non-cancerous reference cell line to provide a general comparison of cytotoxic sensitivity.

### 2.2. Apoptosis Assay

**Figure 2:**
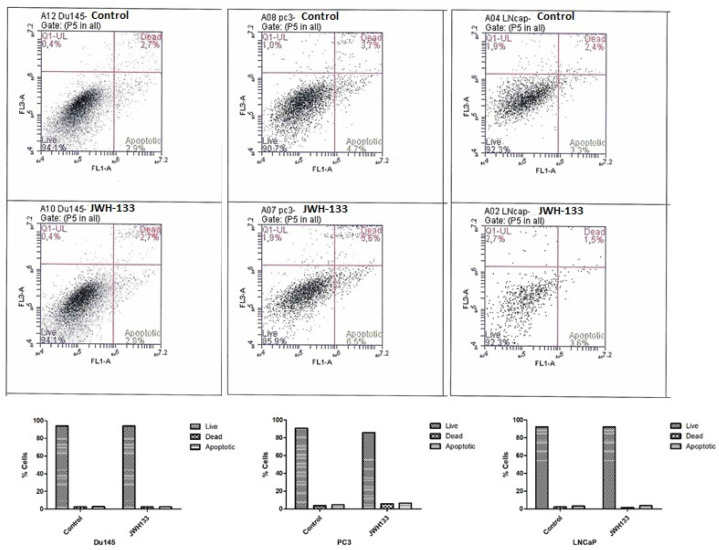
**JWH-133 did not induce apoptosis in prostate cancer cells.** Du-145, PC3, and LNCaP cells were treated for 24 h and 48 h with the indicated concentrations of *JWH-133*. After incubation, cells were trypsinized, washed with ice-cold PBS, suspended in 50 mL of binding buffer, stained with 2.5 mL of FITC Annexin V and 2.5 mL of PI for 15 min in the dark, and then measured by flow cytometer. FACS quantification of the total apoptotic cell population, including dead cells and apoptotic cells. Data are presented as the mean ± SD (*n* = 3).

To determine whether JWH133 induces apoptosis in prostate cancer cells, we next conducted Annexin-V-FITC/PI double staining and flow cytometry analysis. As shown in Figure 2, the apoptotic cell population in Du-145, PC3, and LNCaP cells did not change significantly after JWH133 treatment.

### 2.3. Colony Formation Assay

**Figure 3:**
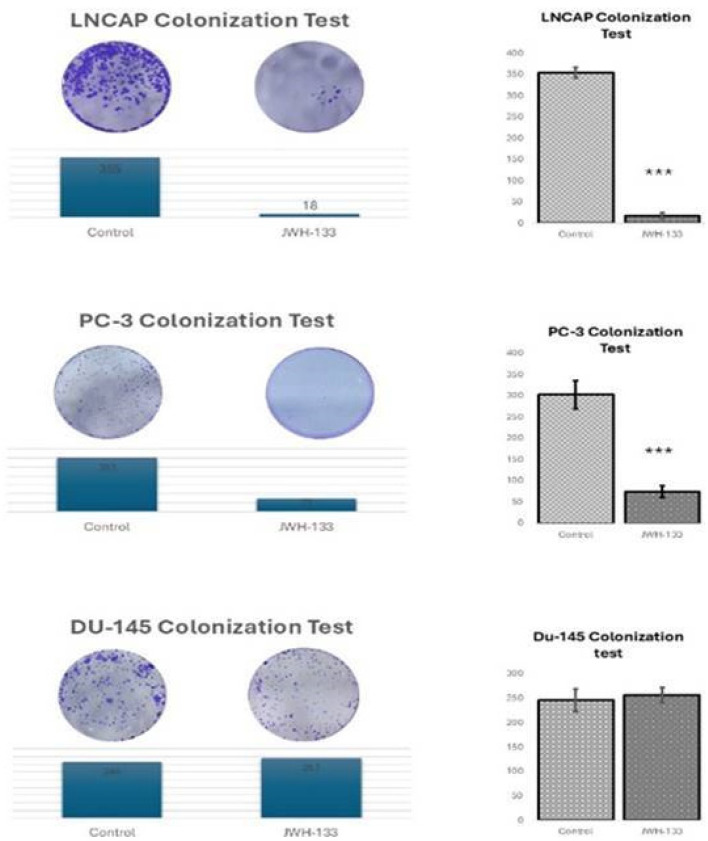
*JWH-133* decreases colony formation in LNCaP and PC-3 prostate cancer cell lines. Colonies were stained with crystal violet. Data are expressed as mean ± SD, n = 3, *** *p* < 0.001.

The effects of *JWH-133* on colony-forming capacity were evaluated in LNCaP, DU-145, and PC3 prostate cancer cell lines. Treatment with *JWH-133* reduced colony formation in PC3 and LNCaP cells, with a more pronounced effect observed in LNCaP cells. In DU-145 cells, the reduction in colony formation was less evident compared with the other prostate cancer cell lines (Figure 3).

### 2.4. Migration Assay

**Figure 4:**
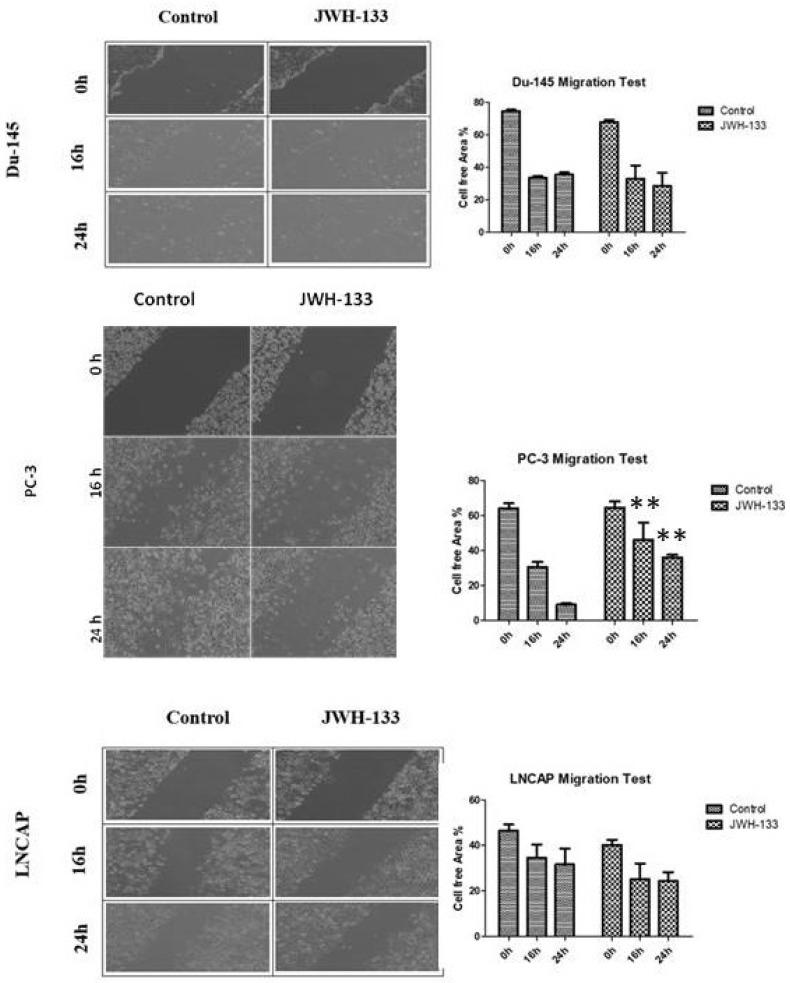
**Wound-healing assay results of prostate cancer cells**. Graph showing migration for both control and dose groups. Control and dose images at 0 h, 16 h, and 24 h were given. Data were presented as mean ± SD. n = 3, ** *p* < 0.01.

In the control groups, the cell-free area progressively decreased over time, reflecting spontaneous wound closure. In DU-145 and LNCaP cells, *JWH-133*-treated groups also showed a time-dependent decrease in cell-free area, suggesting limited inhibition of wound closure. In contrast, PC3 cells maintained a higher remaining cell-free area following *JWH-133* treatment, indicating a more pronounced reduction in wound closure in this cell line (Figure 4).

### 2.5. Real-Time PCR

To investigate the transcriptional effects of *JWH-133* on apoptosis-related pathways, quantitative real-time PCR analyses were performed in DU-145 and PC3 prostate cancer cells. In PC3 cells, *JWH-133* treatment significantly altered the expression levels of several apoptosis- and cell-cycle-related genes, including BAX, BCL-XL, P53, P21, and PUMA (Table 1). In DU-145 cells, significant changes were observed in the expression levels of CASP-3, BCL-XL, P53, P21, PUMA, and NOXA following treatment (Table 2). However, some apoptosis-related genes did not demonstrate statistically significant alterations in either cell line. Overall, these findings indicate that *JWH-133* modulates the transcriptional profile of apoptosis-related genes in a cell-line-dependent manner.

## 3. Discussion

Accumulating research indicates that, in addition to their well-established palliative effects on cancer-related symptoms, cannabinoids can actively suppress tumor growth. This antitumor activity is mediated by the modulation of critical cellular signaling pathways involved in the regulation of cancer cell proliferation and survival. Furthermore, cannabinoids have been shown to inhibit angiogenesis and impair cell proliferation across various tumor types [15]. It has been shown that both natural cannabinoid ligands and synthetic ligands reduce tumor cell proliferation, including that of prostate tumor cells [11,15]. Specifically, endocannabinoids exert potent antiproliferative effects across a wide spectrum of cancer types, including breast, brain, skin, thyroid, prostate, and colorectal carcinomas [16]. For instance, elevated levels of the endocannabinoid 2-arachidonoylglycerol (2-AG) have been reported to inhibit the invasion of PC-3 and DU-145 prostate cancer cells; conversely, suppressing 2-AG synthesis or pharmacologically blocking its binding to CB_1_ receptors with antagonists stimulates cell invasiveness [17]. Furthermore, Olea-Herrero et al. demonstrated that the CB_2_ agonist JWH-015 significantly reduced tumor growth in vivo and exhibited robust antiproliferative effects in PC-3 cells in vitro [11].

Prior literature indicates that the expression levels of both CB_1_ and CB_2_ receptors are significantly elevated in human prostate cancer cells compared to their normal counterparts. Pharmacological administration of a dual CB_1_/CB_2_ agonist has been shown to reduce proliferation, migration, and invasion in prostate cancer cells, while simultaneously inducing apoptosis and triggering cell cycle arrest at the G0/G1 phase. Furthermore, this treatment leads to a marked downregulation in the protein expression of key oncogenic markers, including prostate-specific antigen (PSA), androgen receptor (AR), proliferating cell nuclear antigen (PCNA), and vascular endothelial growth factor (VEGF) [10,13].

The selective CB_2_ agonist *JWH-133* has been demonstrated to exert potent antiproliferative and antiangiogenic effects in non-small cell lung cancer (NSCLC) cells in vitro [18]. Consistently, Preet et al. reported that *JWH-133* effectively inhibits tumor growth and lung metastasis in NSCLC models [18]. Beyond lung malignancies, *JWH-133* has been shown to induce apoptosis in the breast cancer cell line MDA-MB-231 in vitro, while concurrently suppressing cell proliferation, migration, and significantly reducing tumor progression and metastasis in vivo [19]. Furthermore, independent in vivo investigations confirmed that *JWH-133* administration markedly decreases breast tumor burden, lung metastases, and tumor-associated angiogenesis, thereby slowing tumor growth and promoting apoptotic cell death [20].

Notably, CB_2_ receptor expression levels in astrocytomas have been directly correlated with tumor malignancy, with the administration of *JWH-133* completely halting the growth of stage IV human astrocytoma cells in murine models [21]. Similarly, local application of *JWH-133* has been demonstrated to attenuate angiogenesis, restrict tumor growth, and stimulate apoptosis in non-melanoma skin cancer in vivo [22].

Although *JWH-133* modulated apoptosis-related gene expression, Annexin V/PI staining revealed no significant induction of phenotypic cell death in prostate cancer cells. This discrepancy suggests that the observed transcriptional changes were insufficient to trigger the final apoptotic cascade. Instead, our functional assays demonstrated that *JWH-133* profoundly suppressed cell migration and colony formation. Rather than acting as a direct cytotoxic drug at the tested concentrations, these findings indicate that *JWH-133* primarily impairs the aggressive and metastatic potential of cancer cells, serving as a promising anti-invasive therapeutic candidate.

In the present study, *JWH-133* treatment significantly suppressed wound closure in PC-3 cells, whereas DU-145 and LNCaP cells demonstrated more limited migratory sensitivity under the tested experimental conditions. These findings suggest that the biological efficacy of *JWH-133* varies across prostate cancer cell lines possessing distinct molecular profiles. Furthermore, the differential phenotypes observed between androgen-dependent and androgen-independent lines may reflect variations in downstream signaling pathways, receptor expression landscapes, or intrinsic proliferative behaviors. The differential effects of *JWH-133* on migration and gene expression may be attributed to the distinct molecular characteristics of the prostate cancer cell lines, including androgen sensitivity, AR signaling, p53 status, and PTEN alterations. LNCaP cells are androgen-sensitive and AR-positive, whereas PC-3 and DU145 cells are androgen-independent. Moreover, PC-3 cells are PTEN- and p53-deficient, while DU145 cells harbor mutant p53. Consistent with these molecular differences, *JWH-133* significantly inhibited migration only in PC-3 cells. qPCR analysis also revealed increased TP53 expression in PC-3 cells, whereas DU145 cells exhibited decreased TP53 and increased CDKN1A (p21) expression, suggesting that *JWH-133* may activate distinct molecular pathways depending on the genetic background of each cell line. Consequently, further mechanistic investigations are warranted to elucidate the precise molecular underpinnings of these cell-line-specific responses.

The differential gene expression patterns observed between PC3 and DU-145 cells suggest that cellular responses to *JWH-133* may vary depending on the molecular characteristics of each prostate cancer cell line. While several apoptosis- and cell-cycle-related genes demonstrated altered expression following treatment, the biological significance of these transcriptional changes requires further investigation at the protein and functional levels.

The antitumor effects of cannabinoids go beyond classical apoptosis and cell cycle regulation, triggering a more complex signaling network that encompasses regulated cell death (RCD) and metabolic intervention pathways. Recent findings demonstrate that natural compounds with cannabinoid-like effects render cancer cells susceptible to death, particularly by inhibiting PI3K/AKT/mTOR signaling and triggering ferroptosis via the p53/GPX4/SLC7A11 axis. This is directly consistent with cannabinoids’ ability to disrupt the redox balance in cancer cells and increase lipid peroxidation [23]. Furthermore, a study emphasize that forms of inflammatory cell death, such as pyroptosis, and the enhancement of these processes through autophagy inhibition, hold strategic importance in boosting tumor immunity. The multifaceted effects of cannabinoids on autophagy and metabolic reprogramming offer a potential therapeutic foundation for targeting these new-generation RCD pathways and open the door to a synergistic approach in future cancer treatments [24].

Furthermore, a study demonstrates that cannabinoids exhibit a multidimensional antitumor effect by targeting metabolic reprogramming in cancer cells. It appears that cannabinoids disrupt the biosynthetic and energy requirements of cancer cells, particularly by inhibiting critical pathways such as glycolysis (the Warburg effect), the pentose phosphate pathway, and fatty acid synthesis. These compounds trigger oxidative stress and mitochondrial damage by increasing ROS production, which in turn leads to apoptosis via caspase activation. At the same time, they induce metabolic stress in cancer cells through AMPK activation and inhibition of the PI3K/Akt/mTOR signaling pathway, thereby triggering cell cycle arrest and autophagy mechanisms. In particular, their effects on key regulators such as p53 and HIF-1α disrupt the tumor’s adaptation to the hypoxic microenvironment, thereby reducing its metastatic potential and drug resistance [25].

Taken together, the present findings indicate that *JWH-133* influences multiple phenotypic responses in prostate cancer cells, including viability, colony formation, migration-related behavior, and transcriptional regulation of selected genes. However, the underlying molecular mechanisms responsible for these effects remain unclear. In particular, the contribution of CB_2_ receptor-mediated signaling pathways requires further investigation through receptor expression analyses and protein-level functional studies.

Several limitations of the present study should be acknowledged. First, CB_2_ receptor expression levels were not directly evaluated in the prostate cancer cell lines used in this study. In addition, protein-level analyses were not performed to confirm the functional significance of the observed transcriptional alterations. Furthermore, the use of MRC-5 cells as a non-cancerous reference model may not fully reflect the biological characteristics of normal prostate epithelial cells. Therefore, further investigations incorporating receptor-level characterization, protein-based functional assays, and non-malignant prostate epithelial cell models are warranted to fully elucidate the biological efficacy of *JWH-133* in prostate cancer. The other limitations are the exclusively in vitro design, lack of in vivo validation, absence of pharmacokinetic considerations, lack of long-term resistance studies and absence of combination therapy investigations In addition, although *JWH-133* is widely recognized as a highly selective CB_2_ receptor agonist, the present study did not directly evaluate CB_2_ receptor expression in the prostate cancer cell lines or investigate receptor dependency using pharmacological antagonists or genetic approaches. Therefore, the observed antiproliferative and pro-apoptotic effects cannot be conclusively attributed to CB_2_ receptor activation, and the possibility of CB_2_-independent or off-target mechanisms cannot be excluded. These findings should therefore be interpreted with caution. Future studies should determine CB_2_ receptor expression in the experimental models and confirm receptor involvement through the use of selective CB_2_ antagonists, such as AM630, or genetic knockdown/knockout strategies. Such experiments will be essential to establish whether the biological effects of *JWH-133* are mediated specifically through CB_2_ receptor signaling and to further clarify the underlying molecular mechanisms.

Further empirical evidence comes from Zhang et al., who found that treatment with *JWH-133* induced cell death in breast cancer cell lines such as MDA-MB-231 and enhanced the efficacy of other therapeutic approaches, including photodynamic therapy, pointing to a synergistic effect that may be beneficial for treatment [26]. Similarly, Caffarel et al. noted that cannabinoids could reduce the progression of breast cancer driven by the ErbB2 oncogene through Akt signaling inhibition, highlighting a critical pathway in tumorigenesis [20].

Interestingly, the effects of *JWH-133* may vary based on the cancer model. Singh et al. reported that the interaction of cannabinoid treatment with opioid medications can influence pain and cancer progression; however, the specific anti-cancer properties of *JWH-133* remain significant [27]. Although Aso et al.’s work focused primarily on neuroprotection through CB_2_ activation in models of neurodegeneration, it suggests some overlap between cannabinoid receptor activation and pathways of cancer biology [28].

Conflict arises from the findings by Blanton et al., which indicate that *JWH-133* could promote tumor growth under certain conditions, particularly when used in immunocompromised models. Their study revealed an increase in ectopic ovarian tumor growth with *JWH-133* administration, suggesting the context of use and immune system status can significantly affect outcomes associated with cannabinoid therapy [29]. This highlights the necessity for further research to clarify the precise effects and interactions of *JWH-133* across various cancer types and biological contexts.

In conclusion, the present study demonstrates that *JWH-133* influences several cellular responses in prostate cancer cell lines, including cell viability, colony-forming capacity, migration-related behavior, and transcriptional regulation of selected genes. The observed effects differed among prostate cancer cell lines, suggesting cell-line-dependent responses to *JWH-133* treatment. However, the precise molecular mechanisms underlying these effects remain unclear and require further investigation through receptor-level and protein-based functional analyses.

## 4. Materials and Methods

### 4.1. Cell Lines and Reagents

The human prostate cancer cell lines DU-145, PC-3, and LNCaP obtained from the American Type Culture Collection ATCC, Manassas, VA, USA. were utilized in this study. Cells were maintained using RPMI-1640 medium supplemented with L-glutamine (NutriCulture, Preston, UK), 10% heat-inactivated fetal bovine serum (FBS; Biosera, Nuaille, France), and 1% penicillin–streptomycin (Gibco, Grand Island, NY, USA). Additional reagents included 0.25% trypsin-EDTA (Gibco, Grand Island, NY, USA), TriPure Isolation Reagent for RNA/DNA/protein extraction (Roche Diagnostics, Mannheim, Germany), phosphate-buffered saline (PBS; Grisp, Porto, Portugal), MTT reagent (Calbiochem, San Diego, CA, USA), and the iScript^TM^ cDNA synthesis kit (Bio-Rad Laboratories, Hercules, CA, USA).

### 4.2. Cell Culture

Cells were cultured in RPMI-1640 medium containing 10% FBS and 1% penicillin–streptomycin. They were maintained at 37 °C in a humidified incubator with 5% CO_2_ and 90% relative humidity. Culture medium was refreshed every 48 h, and cells were subcultured once optimal confluency was achieved.

### 4.3. Cell Viability and Cytotoxicity Assay

Cell viability and cytotoxicity were evaluated using the MTT assay. The non-cancerous human lung fibroblast cell line MRC-5 was included as a non-malignant reference control to assess differential cytotoxic sensitivity. DU-145, PC-3, LNCaP, and MRC-5 cells were seeded into 96-well plates at a density of 1 × 10^4^ and incubated overnight to allow for cell attachment. Subsequently, the cells were treated with varying concentrations of *JWH-133* (16–192 μM) for 24, 48, and 72 h. This concentration range was selected based on preliminary dose–response experiments and established literature investigating cannabinoid receptor agonists in cancer models [11,13]. Following treatment, culture media were replaced with 5 mg/mL MTT solution and incubated for 3–4 h. The resulting formazan crystals were dissolved in 100 μL DMSO, and absorbance was measured at 570 nm using a microplate reader. IC_50_ values for *JWH-133* were determined from dose–response curves generated using nonlinear regression analysis following cell viability assays. Data were analyzed in GraphPad Prism 9, and IC_50_ values were calculated for subsequent experiments.

### 4.4. Annexin V Apoptosis Assay

Apoptosis in cultured cell lines was assessed using the Annexin V-FITC/Propidium Iodide Staining method. Briefly, cells were harvested, washed twice with cold PBS, and resuspended in binding buffer. Subsequently, cells were incubated with Annexin V-FITC and propidium iodide (PI) for 10–15 min at room temperature in the dark. Samples were analyzed by Flow Cytometry, and apoptotic cell populations were determined based on Annexin V and PI staining profiles.

### 4.5. Colony Formation Assay

Cells were seeded in 6-well plates (1000 cells/well) and allowed to adhere overnight. Cultures were then treated with *JWH-133* at the predetermined IC_50_ concentrations. Medium was replaced with fresh drug-containing medium every 48 h for 10 days. Colonies were fixed, stained with crystal violet, and quantified using ImageJ 1.54d software.

### 4.6. Wound Healing Migration Assay

The effect of *JWH-133* on cell migration was assessed using a wound healing assay. Migration experiments were performed using the IC_50_ concentrations determined from MTT analysis in order to evaluate the overall cellular response to *JWH-133* treatment. DU-145, PC-3, and LNCaP cells were seeded into 6-well plates at a density of 3 × 10^5^ cells/well and cultured until ~85% confluency. A scratch was introduced across the monolayer using a sterile 10 μL pipette tip. Images were taken at 0, 16, and 24 h, and wound closure was quantified with ImageJ software. All experiments were performed in triplicate.

### 4.7. mRNA Isolation and Quantitative Real-Time PCR

To examine the impact of *JWH-133* on oncogenes and apoptosis-related genes, DU-145, PC-3, and LNCaP cells were seeded in 6-well plates (3 × 10^5^ cells/well) and treated at IC_50_ concentrations. qPCR analyses were performed in DU-145 and PC3 cells, which demonstrated more pronounced responses to *JWH-133* treatment in preliminary functional assays. After 72 h, cells were harvested, and total RNA was extracted using either the NORGEN RNA isolation kit or TriPure Isolation Reagent (Roche, Indianapolis, IN, USA), following the manufacturer’s protocols. RNA concentration and purity were determined using a NanoDrop spectrophotometer by measuring the A260/A280 and A260/A230 ratios. Complementary DNA (cDNA) was synthesized with the iScript^TM^ cDNA synthesis kit (Bio-Rad, Hercules, CA, USA). qPCR reactions were performed using the FastStart Essential DNA Green Master kit (Roche, Mannheim, Germany) on a Bio-Rad real-time PCR system, with GAPDH serving as the housekeeping gene. Primer specificity was confirmed by melt curve analysis, and primer efficiencies were evaluated using standard curves and were within the acceptable range (90–110%). Relative expression levels were calculated using the ΔΔCt method. All experiments were performed using at least three independent biological replicates, with each biological replicate analyzed in triplicate as technical replicates.

### 4.8. Statistical Analysis

Statistical analyses were performed using GraphPad Prism version 9.0 (GraphPad Software, Boston, MA, USA). Data are presented as mean ± standard deviation (SD) from at least three independent experiments. IC_50_ values were calculated using nonlinear regression analysis. Comparisons between groups were performed using two-way ANOVA followed by appropriate post hoc Bonferroni analysis. A *p*-value < 0.05 was considered statistically significant.

## Figures and Tables

**Table 1:** Relative gene expression levels (fold regulation) and *p*-values of apoptosis—related genes in cell line PC3 following treatment.

Gene	Fold Regulation	*p*-Value
GAPDH	1.00	NaN
CASP-9	−1.05	0.567314
BAX	1.91	0.013701
BCL-XL	1.27	0.032620
P53	26.42	0.000199
P21	3.62	0.000071
PUMA	−3.93	0.000520

**Table 2:** Relative gene expression levels (fold regulation) and *p*-values of apoptosis—related genes in cell line DU-145 following treatment.

Gene	Fold Regulation	*p*-Value
GAPDH	1.00	NaN
BCL-XL	−1.29	0.000935
P53	−2.44	0.000033
P21	2.78	0.000850
PUMA	1.16	0.000459
NOXA	1.45	0.044368
CASP-3	1.82	0.003648

## Data Availability

Data is contained within the article.
